# Reply to Betsch and Sprengholz: Higher SARS-CoV-2 infection numbers related to more airborne pollen, regardless of testing frequency

**DOI:** 10.1073/pnas.2110982118

**Published:** 2021-08-16

**Authors:** Stefanie Gilles, Claudia Traidl-Hoffmann, Athanasios Damialis

**Affiliations:** ^a^Department of Environmental Medicine, Faculty of Medicine, University of Augsburg, 86156 Augsburg, Germany;; ^b^Institute of Environmental Medicine, Helmholtz Center Munich–Research for Environmental Health, 86156 Augsburg, Germany

Betsch and Sprengholz (1) address the issue of supposedly increased severe acute respiratory syndrome coronavirus-2 (SARS-CoV-2) testing frequency in allergic patients during the pollen season because of the misinterpreted similar symptoms of allergic and viral rhinitis. We (2) had previously reported a significant and positive relationship between SARS-CoV-2 infections and airborne pollen concentrations in early Spring 2020, with potential mechanism of a previously discovered immunosuppressive effect of pollen on the nasal epithelium (3), possibly more pronounced in allergics due to a known intrinsic reduction of antiviral type I and type III interferon responses (4, 5).

In this study (1), several parameters may have influenced the conclusion drawn. First, “allergic” participants were identified based on self-reported “hay fever” (yes/no) without further stratification for the type of pollen allergy; it is therefore probable that among them a proportion might be responsive to other pollen types than the relevant tree pollen for the examined season.

Second, the authors used airborne pollen data from across Germany, based on the open-access database https://pollenscience.eu/, using “allergy-relevant” pollen thresholds (30 and 100 m^−3^), whose own creator has highlighted that such thresholds may be disputable (6).

**Fig. 1.**
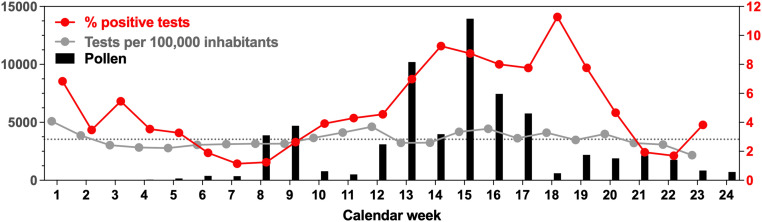
Percentages of positive SARS-CoV-2 tests, but not numbers of total performed tests, coincide with pollen peaks in 2021 in Augsburg, Germany. The study period was January 1 to June 14, 2021. The gray dotted line represents the average weekly number of SARS-CoV-2 tests over the entire study period. Pollen (black bars) is given as weekly averages.

Third, based on their data and analysis code (https://osf.io/j5g7n/) (1), the variable of pollen was not at all included in their models, which means that the magnitude of the pollen effect on infections was not calculated. Moreover, for 2 out of 6 d on which the data were obtained, responses on the PCR test were missing, as on April 20, which was within the peak of the birch pollen season in Augsburg (Fig. 1), as well as in other locations in Germany (https://pollenscience.eu/).

**Fig. 2.**
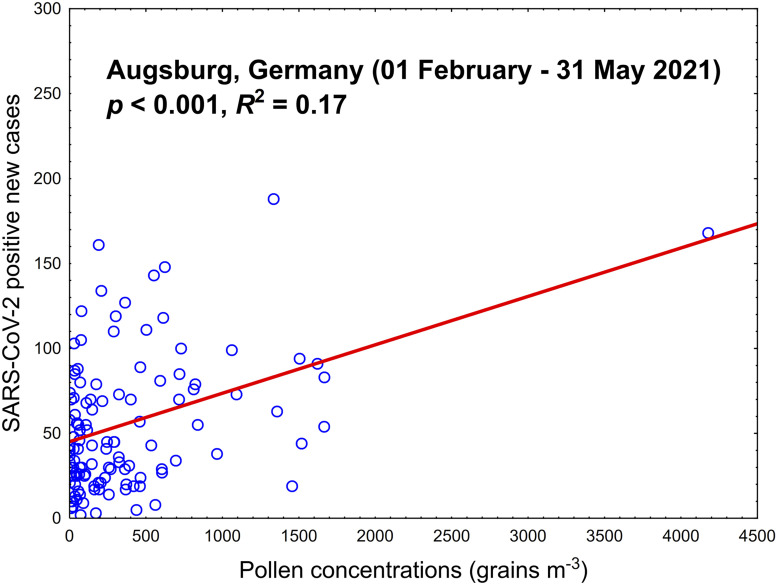
Scatterplot (general linear model, simple regression) of SARS-CoV-2–positive new cases vs. airborne pollen concentrations, controlled for the number of COVID-19 tests. The study period was selected based on the first consistent higher pollen concentrations (February 1 to May 31, 2021).

We do acknowledge with Betsch and Sprengholz (1) that it is indeed important to control for testing; hence, since January 1, 2021, we have collected weekly numbers of SARS-CoV-2 tests, as publicly available from the city of Augsburg, Germany (https://www.augsburg.de/umwelt-soziales/gesundheit/coronavirus/fallzahlen; last access: June 17, 2021), as well as pollen measurements (https://wiki.unika-t.de/tiki-index.php?page=Pollenflug; last access: June 17, 2021). We found that while the number of tests per week remained relatively stable, the percentage of positive tests peaked between weeks 13 and 19 (Fig. 1). The peak in positive, not total, tests coincided with the local pollen peak. Finally, we plotted (general linear models, simple regression) the daily infection numbers and pollen concentrations (February 1 to May 31, 2021), controlled for the number of tests performed; we found a positive relationship (*P* < 0.001, *R*^2^ = 0.17; Fig. 2). When not controlling for the number of tests performed, the same relationship arose (*P* < 0.001), with a minor decrease in *R*^2^ = 0.16.

Overall, we agree that the testing is a significant parameter to be taken into account when modeling the spreading of COVID-19, but when integrated into other more significant variables, like airborne pollen concentrations, the magnitude of difference in the explained variability is minimal, only 1%.
